# Evolution of gastric surgery techniques and outcomes

**DOI:** 10.1186/s40880-016-0134-y

**Published:** 2016-07-26

**Authors:** Hironori Shiozaki, Yusuke Shimodaira, Elena Elimova, Roopma Wadhwa, Kazuki Sudo, Kazuto Harada, Jeannelyn S. Estrella, Prajnan Das, Brian Badgwell, Jaffer A. Ajani

**Affiliations:** 1 Department of Gastrointestinal Medical Oncology, Unit 426, The University of Texas MD Anderson Cancer Center, 1515 Holcombe Blvd, Houston, TX 77030 USA; 2 National Cancer Center Hospital, Tokyo, 104-0045 Japan; 3 Department of Pathology, The University of Texas MD Anderson Cancer Center, Houston, TX 77030 USA; 4 Department of Radiation Oncology, The University of Texas MD Anderson Cancer Center, Houston, TX 77030 USA; 5 Department of Surgical Oncology, The University of Texas MD Anderson Cancer Center, Houston, TX 77030 USA

**Keywords:** Gastric cancer, Gastrectomy, Laparoscopic gastrectomy, Lymph node dissection

## Abstract

Surgical management of gastric cancer improves survival. However, for some time, surgeons have had diverse opinions about the extent of gastrectomy. Researchers have conducted many clinical studies, making slow but steady progress in determining the optimal surgical approach. The extent of lymph node dissection has been one of the major issues in surgery for gastric cancer. Many trials demonstrated that D2 dissection resulted in greater morbidity and mortality than D1 dissection. However, long-term outcomes demonstrated that D2 dissection resulted in longer survival than D1 dissection. In 2004, the Japan Clinical Oncology Group reported a pivotal trial which was performed to determine whether para-aortic lymph node dissection combined with D2 dissection was superior to D2 dissection alone and found no benefit of the additional surgery. Gastrectomy with pancreatectomy, splenectomy, and bursectomy was initially recommended as part of the D2 dissection. Now, pancreas-preserving total gastrectomy with D2 dissection is standard, and ongoing trials are addressing the role of splenectomy. Furthermore, the feasibility and safety of laparoscopic gastrectomy are well established. Survival and quality of life are increasingly recognized as the most important endpoints. In this review, we present perspectives on surgical techniques and important trials of these techniques in gastric cancer patients.

## Background

Gastric cancer (GC) is an aggressive malignancy. In 2012, according to the World Health Organization GLOBOCAN database, it affected 952,000 people and resulted in 723,000 deaths [1]. Although the death rate for GC is high, it has decreased gradually over the past few decades [2]. GC is common in Asia, South America, and Central and Eastern Europe but uncommon in other parts of Europe, North America, and most parts of Africa [1, 3]. GC is a common cancer in Japan, with higher overall mortality than that in other countries [4–7]. Thus, owing to extensive experience in treating GC, Japanese surgeons have been leading the surgical management of GC and recommend extended lymph node dissection. In 2001, physicians in Japan established guidelines for the treatment of GC, along with the extent of lymph node dissection. These guidelines have undergone occasional revision, with the latest English version published in 2013 [8]. This review focuses on dissection of lymph nodes, resection of organs surrounding the stomach, and laparoscopic surgery in GC patients.

## Definition of lymph node dissection

According to National Comprehensive Cancer Network guidelines (version 2.2013), “D1 dissection entails gastrectomy and resection of both the greater and lesser omenta (which would include the lymph nodes along right and left cardiac, along lesser and greater curvature, suprapyloric along the right gastric artery, and infrapyloric areas). The D2 dissection would include D1 nodes and all nodes along the left gastric artery, common hepatic artery, celiac artery, splenic hilum, and splenic artery.” [9]. D3 surgery additionally dissects D1 and D2 lymph nodes along with lymph nodes in the hepatoduodenal ligament and retropancreatic region and surrounding the superior mesenteric vein.

## Lymph node dissection

The Medical Research Council in the United Kingdom conducted a prospective multicenter randomized controlled trial (RCT) with 200 patients in each arm who underwent D1 or D2 dissection and total or subtotal gastrectomy and reported the results in 1996 [10]. Postoperative morbidity (46% vs. 28%, *P* < 0.001) and mortality (13.0% vs. 6.5%, *P* = 0.04) were higher in the D2 group than in the D1 group [10]. The follow-up data demonstrated no difference in the overall survival (OS) rate between the two groups (35% vs. 33%, *P* = 0.43) [11].

In 1995 the Dutch Gastric Cancer Group performed a prospective multicenter RCT with 711 patients who underwent D1 or D2 node dissection (380 in the D1 arm and 331 in the D2 arm) and gastrectomy [12]. The D2 group had higher postoperative morbidity and mortality and longer hospitalization times than the D1 group. However, the 5-year OS rates were similar (45% for the D1 group and 47% for the D2 group, *P* = 0.99) [13]. After 11 years of follow-up, the OS rates did not differ significantly between the D1 and D2 groups (30% vs. 35%, *P* = 0.53) [14]. 15-year follow-up analysis demonstrated markedly more GC-related deaths in the D1 group; in addition, local and regional recurrence rates were higher in the D1 group than in the D2 group (22% vs. 12% and 19% vs. 13%, respectively) [15].

In 1994, the Italian Gastric Cancer Study Group conducted a phase II RCT of D1 and D2 dissection in GC patients [16, 17]. It demonstrated postoperative morbidity and mortality in the D2 and D1 groups (20.9% and 3.1%, respectively) similar to those reported previously [15]. In that trial, as opposed to prior trials, the investigators did not perform pancreatectomy. Based on these results, the Italian Gastric Cancer Study Group performed another RCT to compare gastrectomy with D1 and D2 dissection and reported the short-term results in 2010, which demonstrated no significant differences in postoperative morbidity or mortality between the two groups [18]. Follow-up analysis demonstrated no differences in the 5-year OS rate [19]. In subgroup analyses, the D1 group had a higher 5-year disease-specific survival rate in patients with pathologic T1 disease than the D2 group (98% vs. 83%, *P* = 0.015), whereas the D2 group had a higher 5-year disease-specific survival rate in patients with pathologic T2-4 disease and cancer-positive lymph nodes than the D1 group (59% vs. 38%, *P* = 0.055) (Table 1). However, such ad hoc results are not reliable.

**Table 1:** Randomized trials of lymph node dissection in patients with gastric cancer: D1 vs. D2

References	Dissection type	No. of patients	Morbidity (%)	Mortality (%)	5-year OS rate (%)	15-year follow-up (%)		
						OS rate	Local RR	Regional RR
Cuschieri et al. [10, 11]	D1	200	27.5	6.5	35.0	–	–	–
	D2	200	46.0	13.0	33.0	–	–	–
Bonenkamp et al. [12–14]	D1	380	24.7	3.9	45.0	21.6	21.6	19.2
	D2	331	42.9	9.7	47.0	27.8	12.1	13.0
Degiuli et al. [18, 19]	D1	133	12.0	3.0	66.5	–	–	–
	D2	134	17.9	2.2	64.2	–	–	–

– no data available, *OS* overall survival, *RR* recurrence rate

In 2006 researchers in Taiwan, China conducted a single-center RCT comparing D1 and D3 dissections in combination with gastrectomy [20]. They randomly assigned 221 eligible patients to D1 or D3 dissection performed by 11 specially trained surgeons, each of whom had performed at least 25 independent D3 dissections. D3 dissection resulted in a significantly higher 5-year OS rate than D1 dissection (59.5% vs. 53.6%, *P* = 0.041), although the morbidity was higher in the D3 group. A follow-up study demonstrated that the quality of life did not differ between the two groups [21]. The authors concluded that D3 dissection performed by an experienced surgeon may offer a survival benefit for patients with GC. However, this conclusion contradicts the 2010 Japanese GC treatment guidelines (version 3) [6].

In comparison, the Japan Clinical Oncology Group (JCOG) conducted an RCT (JCOG9501) comparing the outcomes between the two groups treated with gastrectomy plus D2 dissection alone and gastrectomy plus both D2 and para-aortic nodal dissections and reported the results in 2004 [22]. In their study, 523 eligible patients underwent the surgery performed by experienced surgeons. The follow-up results demonstrated no difference in 5-year OS rate (69.2% for the D2 group vs. 70.3% for the D2 and para-aortic nodal dissection group, *P* = 0.85) or recurrence-free survival rate (62.6% for the D2 group vs. 61.7% for the D2 and para-aortic nodal dissection group, *P* = 0.56) [23].

## Splenectomy and pancreatectomy

The purpose of gastrectomy with splenectomy or pancreatectomy along with D2 dissection is the performance of comprehensive surgery. Whether to perform splenectomy and pancreatectomy in GC patients has long been a subject of debate. In 1999, the Medical Research Council conducted a multivariate analysis showing that pancreaticosplenectomy was independently associated with poor survival (hazard ratio 1.53, 95% confidence interval 1.17–2.01) but that splenectomy was not (hazard ratio 1.36, 95% confidence interval 0.97–1.90) [11]. Also, a Dutch Gastric Cancer Group trial published in 2004 suggested that D2 dissection, splenectomy, pancreatectomy, and older patient age (>70 years) were associated with high morbidity and mortality [14].

Investigators in Japan conducted an RCT comparing total gastrectomy plus D2 lymph node dissection with and without pancreatectomy in 2004 [24]. They randomized 110 patients equally to two groups: one group underwent total gastrectomy with removal of the pancreatic body and tail as well as the spleen; the other group underwent total gastrectomy with splenectomy. Although the 5-year OS rates in the two groups did not differ significantly, 6% (1 of 18) of the patients in the pancreatectomy group had diabetes mellitus, 33% (6 of 18) of whom were diagnosed as having impaired glucose tolerance 1 year after surgery, which occurred with markedly higher frequency compared with those in the group without pancreatectomy.

In a prospective RCT comparing total gastrectomy with and without splenectomy in 187 patients in Chile in 2002 [25], 90 patients underwent total gastrectomy with D2 dissection and splenectomy, whereas 97 patients did so without splenectomy. The mortalities for those who underwent D2 dissection with and without splenectomy were not significantly different (3.1% vs. 4.4%, *P* > 0.7). Also, the morbidity was higher in the patients treated with splenectomy than in those without [fever higher than 38 degree, 50% vs. 39% (*P* < 0.04); pulmonary complications, 39% vs. 24% (*P* < 0.08); and subphrenic abscess, 11% vs. 4% (*P* < 0.05)]. The 5-year OS rate did not differ significantly in patients undergoing D2 dissection with and without splenectomy (42% vs. 36%, *P* > 0.5). The authors stated that splenectomy was not necessary.

In 2006, Korea researchers conducted a single-center RCT of gastrectomy with and without splenectomy [26]. They randomly assigned 207 eligible patients with resectable GC to splenectomy (104 patients) and spleen-preserving (103 patients) groups. No significant differences in the postoperative morbidity and mortality were observed between the splenectomy group and the spleen-preserving group [15.4% vs. 8.7% (*P* = 0.142) and 1.9% vs. 1.0% (*P* = 1.000), respectively]. The 5-year OS rate was not significantly higher in the splenectomy group than in the spleen-preserving group (54.8% vs. 48.8%, *P* = 0.503).

Based on these results, in 2002, a multicenter RCT in Japan to determine whether gastrectomy without splenectomy is suitable as a standard surgical treatment of GC (JCOG0110) has completed accrual, and the results are pending.

## Bursectomy

A bursectomy is a dissection of the peritoneal lining covering the pancreas and the anterior aspect of the transverse mesocolon. In 1980s, physicians in Japan performed bursectomy with radical gastrectomy and extended dissection, which meant complete resection of the post-gastric cavity lining and may have included free cancer cells and/or micrometastases [27]. However, it increased the risk of surgical complications. Therefore, according to the guidelines of the Japanese Gastric Cancer Association, bursectomy is recommended only for GC with serosal invasion.

In 2012, the interim results of an RCT regarding the survival benefit of bursectomy were published [28]. Two hundred ten patients with resectable GC were registered and underwent total or distal subtotal gastrectomy and D2 dissection as standard treatment. One hundred four patients underwent the standard procedure with bursectomy, whereas 106 patients underwent the standard procedure without bursectomy. The overall morbidity (14.30%) and mortality (0.95%) were the same in both groups. The difference in 3-year OS rate was not statistically significant between the two groups with and without bursectomy (85.6% vs. 79.6%, *P* = 0.443). Also, the difference in 3-year OS rate was not statistically different between 48 patients with serosa-positive GC treated with and without bursectomy (69.8% vs. 50.2%, *P* = 0.043). However, patients who did not undergo bursectomy had more peritoneal recurrences than patients who did undergo bursectomy (13.2% vs. 8.7%). Long-term results showed that the 5-year OS rates were 77.5% and 66.6% in the bursectomy and non-bursectomy groups, respectively (two-sided, *P* = 0.16 for superiority; one-sided, *P* = 0.99 for noninferiority). The final results of the analysis did not demonstrate the noninferiority of the standard procedure without bursectomy. Therefore, the JCOG is conducting a large multicenter RCT to evaluate bursectomy in patients with T3 (subserosal) or T4 (serosal) GC (JCOG1001), which aims to recruit 1200 patients who will undergo gastrectomy and D2 dissection with or without bursectomy followed by chemotherapy. The primary endpoint is OS; the secondary endpoints are recurrence-free survival, blood loss, operation time, morbidity, mortality, and the rate of adverse effects of adjuvant chemotherapy. The results of this trial are eagerly awaited.

## Laparoscopic surgery

Laparoscopic gastrectomy has been popular since 2000s, and some RCTs assessed the benefit of the laparoscopic procedure. For example, investigators in Japan performed an RCT comparing laparoscopy-assisted distal gastrectomy (LADG) with open distal gastrectomy (ODG) in 2002 [29]. In this study, 28 patients with early-stage GC underwent LADG or ODG with D1 dissection. Patients in the LADG group had less blood loss but longer operation time than the ODG group. The number of lymph nodes examined was not significantly different between the LADG and ODG groups [20.2 vs. 24.9, *P* = not significant (NS)]. Furthermore, bowel function and ambulation of patients in the LADG group recovered earlier than those in the ODG group (bowel function, 1.8 vs. 2.6 days, *P* < 0.05; ambulation, 2.9 vs. 3.9 days, *P* < 0.05).

In 2005, researchers conducted a similar prospective RCT recruited only 28 patients with GC [30]. The operation time was longer in the LADG group than in the ODG group (378 vs. 235 min, *P* < 0.01), and the postoperative hospital stay was shorter in the LADG group than in the ODG group (12 ± 2 vs. 18 ± 6 days, *P* < 0.01).

At the same time, researchers in Korea conducted a single-center RCT [31]. They randomly assigned 47 patients with early-stage GC to undergo either LADG (*n* = 24) or ODG (*n* = 23). The mean operation time was longer in the LADG group than in the ODG group (319.6 vs. 190.4 min, *P* < 0.001). However, the postoperative pulmonary complication rate was lower in the LADG group than in the ODG group (8% vs. 30%, *P* = 0.045). In comparison, investigators in Italy performed a single-center RCT comparing the feasibility and OS of 59 patients with GC in the LADG and ODG groups [32]. They observed no significant differences between the LADG and ODG groups in the study endpoints, including morbidity (23.3% vs. 27.6%, *P* = NS) and mortality (3.3% vs. 6.9%, *P* = NS). The times from the operation to resumption of oral intake and to discharge were shorter in the LADG group (oral intake: 5.1 vs. 7.4 days, *P* < 0.001; discharge: 10.3 vs. 14.5 days, *P* < 0.001). In addition, the 5-year OS rates (58.9% vs. 55.7%, *P* = NS) and the 5-year disease-free survival rates (57.3% vs. 54.8%, *P* = NS) were similar.

In 2008, an RCT conducted in Korea to assess the quality of life of GC patients after LADG or ODG (COACT 0301) [33] showed that blood loss in the LADG group was lower than that in the ODG group (111.6 vs. 267.2 mL, *P* < 0.05) but that the operation time was longer (378 vs. 235 min, *P* < 0.01) and the number of dissected lymph nodes was smaller (39.0 vs. 45.1, *P* < 0.05) in the LADG group. The LADG group experienced earlier weaning from epidural anesthesia (39.4 vs. 47.8 mL, *P* < 0.001), earlier resumption of oral intake (3.8 vs. 4.1 days, *P* = 0.002), shorter hospital stay (7.2 vs. 8.6 days, *P* < 0.001), and better quality of life according to answers to the European Organisation for Research and Treatment of Cancer QLQ-C30 and QLQ-STO22 quality-of-life questionnaires (*P* < 0.001). The 5-year disease-free survival and OS rates in the two groups were similar [34].

In 2010, a phase III multicenter RCT conducted by the Korean Laparoscopic Gastrointestinal Surgery Study Group (KLASS Trial), which included 342 patients randomized to undergo LADG (179 patients) or ODG (163 patients), showed that the postoperative complication rates were 9% (17/179) in the LADG group and 15% (24/163) in the ODG group (*P* = 0.137), with no significant difference in the morbidity (11.6% vs. 15.1%, *P* = 0.137) or mortality (1.12% vs. 0%, *P* = 0.497) [35].

In Japan, two large RCTs with GC patients based on a prior trial (JCOG0703) are ongoing [36]. One RCT is evaluating the noninferiority of OS for LADG compared with ODG (JCOG0912) [37]. The other is a phase II/III RCT by the Japanese Laparoscopic Surgery Study Group, evaluating morbidity and recurrence-free survival in 500 gastric cancer patients who will be registered and undergo LADG or ODG (JLSSG0901) (Table 2).

**Table 2:** Randomized trials of laparoscopic gastrectomy in gastric cancer patients: ODG vs. LADG

References	Procedure	No. of patients	Blood loss (mL)	Operation time (min)	Morbidity (%)	Mortality (%)	No. of LNs	5-year DFS rate (%)	5-year OS rate (%)
Kitano et al. [29]	ODG	14	258.0	171.0	28.6	0	24.9	–	–
	LADG	14	117.0	227.0	14.3	0	20.2	–	–
Hayashi et al. [30]	ODG	14	489.0	235.0	42.9	0	27.0	–	–
	LADG	14	327.0	378.0	14.2	0	28.0	–	–
Lee et al. [31]	ODG	23	336.4	190.4	43.5	0	38.1	–	–
	LADG	24	294.4	319.6	12.5	0	31.8	–	–
Huscher et al. [32]	ODG	29	391.0	168.0	27.6	6.9	33.4	–	–
	LADG	30	229.0	196.0	23.3	3.3	30.0	–	–
Kim et al. [33, 34]	ODG	82	267.2	235.0	NA	0	45.1	97.6	96.3
	LADG	82	111.6	378.0	NA	0	39.0	98.9	97.6

– no data available, *ODG* open distal gastrectomy, *LADG* laparoscopy-assisted distal gastrectomy, *LNs* lymph nodes, *DFS* disease-free survival, *NA* not applicable

## Conclusions

For some time, the optimal method of node dissection has been a subject of intense debate. However, three RCTs comparing D2 and D1 dissection have provided some consensus. D2 dissection may be more beneficial than D1 dissection. Given the results of the Italian Gastric Cancer Study Group study, selecting GC patients for more extensive surgery may be possible. Surgery beyond D2 dissection is not useful (JCOG9501).

Pancreatectomy should be avoided for GC since pancreas-preserving D2 dissection has been shown to be superior to D2 dissection with pancreatectomy [24]. In the RCTs in Chile and Korea comparing gastrectomy with and without splenectomy, the 5-year OS rates did not differ. These results may encourage performing gastrectomy with D2 dissection that preserves the spleen. Results of a JCOG RCT designed to assess the noninferiority of spleen preservation will be helpful in resolving this issue.

LADG is another advance in GC treatment that produces results similar to those of ODG. Many RCTs have demonstrated lower blood loss with and earlier recovery from LADG than with/from ODG. However, operation time has been longer for LADG than for ODG. In some studies, LADG yielded fewer nodes than ODG. Ongoing studies will settle this issue. Additionally, robot-assisted surgery for GC is becoming prevalent, and many studies of neoadjuvant and adjuvant chemotherapy and radiation therapy are in progress, though without sufficient evidence. In the future, the results of ongoing studies may alter operating procedures (Table 3).

**Table 3:** Ongoing randomized trials of GC surgery techniques

Authors and the trial number	Procedure	Target sample size (cases)	Endpoint
Sasako et al. (JCOG 0110)	Gastrectomy with splenectomy vs. without splenectomy	500	OS
Doki et al. (JCOG 1001)	Gastrectomy with bursectomy vs. without bursectomy	1200	OS
Kim et al. (KLASS 01)	ODG vs. LADG	342	OS
Katai et al. (JCOG 0912)	ODG vs. LADG for early-stage GC	920	OS
Kitano et al. (JLSSG0901)	ODG vs. LADG for advanced-stage GC	500	Incidence, RFS

*RFS* recurrence-free survival, *Incidence* incidence of anastomotic leakage or pancreatic fistula, *GC* gastric cancer, *LADG* laparoscopy-assisted distal gastrectomy, *ODG* open distal gastrectomy

In conclusion, GC surgery techniques have evolved over time. D2 dissection without pancreatectomy (and even with splenectomy) has been embraced as the standard, and further developments will bring about the use of robotics.

## Abbreviations

GC: gastric cancer
RCT: randomized controlled trial
JCOG: Japan Clinical Oncology Group
LADG: laparoscopy-assisted distal gastrectomy
ODG: open distal gastrectomy
